# Antimicrobial activity of phytofabricated silver nanoparticles using *Carica papaya* L. against Gram-negative bacteria

**DOI:** 10.14202/vetworld.2023.1301-1311

**Published:** 2023-06-13

**Authors:** Mbarga Manga Joseph Arsene, Podoprigora Irina Viktorovna, Marukhlenko Alla, Morozova Mariya, Anyutoulou Kitio Linda Davares, Bassa Zacharie Carime, Gizinger Oksana Anatolievna, Yashina Natalya Vyacheslavovna, Zhigunova Anna Vladimirovna, Smolyakova Larissa Andreevna, Vasilieva Elena Aleksandrovna, Butusov Leonid Alekseevich, Borekhova Marina Nikolaïevna, Kezimana Parfait, Vodyashkin Andrey

**Affiliations:** 1 Department of Microbiology V.S. Kiktenko, Medical Institute, RUDN University named after Patrice Lumumba, Moscow, Russia; 2 Research Institute of Molecular and Cellular Medicine, Medical Institute RUDN University named after Patrice Lumumba, Moscow, Russia; 3 Department of Pharmaceutical and Toxicological Chemistry, Medical Institute, RUDN University named after Patrice Lumumba, Moscow, Russia; 4 Department of Food Sciences and Nutrition, National School of Agro-industrial Sciences, University of Ngaoundere, Cameroon; 5 Institute of Innovative Engineering Technologies, RUDN University named after Patrice Lumumba, Moscow, Russia; 6 Department of Agrobiotechnology, Agrarian Institute, RUDN University named after Patrice Lumumba, Moscow, Russia; 7 Institute of Biochemical Technology and Nanotechnology. RUDN University named after Patrice Lumumba, Moscow, Russia

**Keywords:** antibiotic resistance, biogenic synthesis, *Carica papaya*, Gram-negative, silver nanoparticles

## Abstract

**Background and Aim:**

Antibiotic resistance, especially in Gram-negative bacteria, is a major public health risk affecting all industries requiring the use of antibiotics, including agriculture and animal breeding. This study aimed to use papaya extracts to synthesize silver nanoparticles (AgNPs) and evaluate their antimicrobial activity against various Gram-negative bacteria.

**Materials and Methods:**

Silver nanoparticles were synthesized from the aqueous extracts of papaya seed, root, and bark, with AgNO_3_ used as a reducing agent. The phytofabricated AgNPs were analyzed by ultraviolet–visible absorbance, X-ray diffraction (XRD), Fourier-transform infrared spectroscopy, and photon cross-correlation spectroscopy (PCCS). The disc-diffusion method was used to perform antibacterial analysis, and the minimum inhibitory concentrations (MIC) and minimum bactericidal concentrations were determined. We also investigated the antibiofilm activity of AgNPs and attempted to elucidate the potential mechanism of action on *Escherichia coli* ATCC 25922.

**Results:**

Phytofabrication of AgNPs was successful with papaya root (PR-AgNPs) and papaya seed (PS-AgNPs), but not with papaya bark. Silver nanoparticles using papaya root and PS-AgNPs were both cubic and showed maximum absorbances of 2.6 and 0.3 AUs at 411.6 and 416.8 nm wavelengths and average hydrodynamic diameters X50 of 59.46 ± 7.03 and 66.57 ± 8.89 nm, respectively. The Ag in both AgNPs was confirmed by X-ray fluorescence by a distinctive peak in the spectrum at the silver Kα line of 22.105 keV. Both AgNPs exhibited broad-spectrum antimicrobial and antibiofilm activity against all Gram-negative bacteria, and PR-AgNPs were slightly better than AgNPs-PS. The MIC ranged from 16 μg/mL–128 μg/mL and 16 μg/mL–64 μg/mL, respectively, for PS-AgNPs and PR-AgNPs. The elucidation of the mechanism of action revealed interference with *E. coli* ATCC 25922 growth kinetics and inhibition of H^+^-ATPase proton pumps.

**Conclusion:**

Papaya seed and root extracts were efficient reducing agents for the biogenic synthesis of AgNPs, with noteworthy antibacterial and antibiofilm activities. Future studies should be conducted to identify the phytochemicals and the mechanism involved in AgNPs synthesis.

## Introduction

Antibiotic resistance is the ability of bacteria to resist antimicrobials to which they are susceptible [1–3]. This phenomenon is particularly observed in Gram-negative bacteria because of their ability to easily accumulate resistance genes and the presence of efflux pumps in their membranes, which are used to expel antimicrobials from the cells. This capability makes these bacteria unresponsive or resistant to various types of antibiotics [3–5].

In general, antibiotic resistance has prompted a major mobilization in pursuing new antimicrobial compounds and alternative treatments [2]. Several matrices with physiological potentials, such as probiotics [6, 7], medicinal plants [8–10], antimicrobial peptides [11, 12], phages [13, 14], and various nanoparticles (NPs) [3, 15, 16], are regularly suggested as the most credible alternatives to common antibiotics. In recent years, there has also been a considerable increase in research to study the antibacterial activity of metallic nanostructures and nanocomplexes, with silver NPs (AgNPs) being among the most investigated [3, 15–17]. The synthesis of NPs is very often performed by chemical or physical means, but unfortunately, these methods have ecological concerns, hence the need to use more eco-friendly routes [18, 19]. The biogenic synthesis of NPs is a simple and direct route to the formation of NPs using biological materials, such as metabolites from animals, enzymes, microorganisms, and plant extracts [17, 20]. These green pathways, using plant extracts in particular, are highly recommended to replace chemical methods since the resulting NPs seem more stable and cost-effective [17, 21, 22]. Despite this benefit, it is important to highlight that, with NPs synthesized by chemical or physical methods, biosynthesized AgNPs can be toxic to other organisms, such as plants or animals, if not used properly despite their good antimicrobial action [18, 20]. Various plants, medicinal or non-medicinal, such as *Panax ginseng* [20]; *Euphorbia wallichii* [23], *Salvia verticillata* and *Filipendula ulmaria* [24], *Phoenix dactylifera* [25], *Debregeasia salicifolia* [26], *Moringa oleifera* [27], and *Datura metel* [28], have already been used in the phytofabrication and stabilization of AgNPs. Very few studies have investigated the synthesis of AgNPs using *Carica papaya* [29, 30].

*Carica papaya*, known as papaya, papaw, or pawpaw, is a flowering and dicotyledonous plant classified as Violales order, Caricaceae family, *Carica* L. genus, and papaya species (papaya informally) [31]. Papaya trees are mostly cultivated in tropical and subtropical regions and are fast-growing and semi-woody with a lifespan of approximately 5–10 years [32]. Papaya fruits are melon-like, with green skin that turns yellow or orange when ripe and reportedly contain >1000 seeds [31]. Papaya tree parts could be a good candidate for the phytofabrication of AgNPs given their phytochemicals [30]. Indeed, papaya reportedly contains alkaloids, such as carpain and pseudocarpain; enzymes, such as papain and chymopapain; and other compounds, such as benzyl isothiocyanate or benzyl glucosinolate [33, 34]. All of these compounds can have significant roles not only in reducing silver ions (Ag+) to form Ag, but above all as capping and stabilizing element for AgNPs.

This study aimed to evaluate the use of aqueous extracts from *C. papaya* L. (seeds, bark, and leaves) to produce AgNPs and investigate their antibacterial activity against Gram-negative bacteria. The antibiofilm activity of the synthesized AgNPs was also investigated.

## Material and Methods

### Ethical approval

There was no ethical concern and no specific approval was required since all the work was carried out *in vitro*.

### Study period and location

The study was conducted from December 2021 to May 2022. All work was done at the Microbiology Laboratory VS Kiktenko of RUDN University, Moscow, Russia.

### Plant collection

After the identification of the plant using the mobile professional version of PictureThis - Plant Identifier App (Glority LLC, 2021), different parts (papaya seeds [PS], bark and roots) of the plant were collected during a holiday’s trip in December 2021 in the city of Nlobison II (Cameroon). After air drying, the samples were packed and sent to the Microbiology Laboratory of RUDN University.

### Extract preparation

Phytochemical compounds were extracted with water as solvent (270 mL) and 30 g of each vegetal material. The mixture was stirred at 11× *g* at 25°C for 24 h and then filtered through Whatman filter paper № 1.

### Phytofabrication of AgNPs

A 1 mM AgNO_3_ (PanReac AppliChem, Darmstadt, Germany) solution was prepared, and 180 mL of the solution was mixed with 20 mL of each extract. The reaction mixture was incubated in the dark at 42°C for 24 h for bioreduction.

### Purification and characterization of AgNPs

The AgNPs were recovered by centrifuging the reaction mixture for 1 h at 21428× *g*, and the pellet was washed with ethanol (99%) and then with distilled water (4 times) [17]. The pellet was finally dried at 40°C until complete drying. The AgNPs were characterized by ultraviolet (UV)-visible spectrophotometry (PerkinElmer Lambda 950 spectrophotometer) from 350 to 800 nm, photon cross-correlation spectroscopy (PCCS) (Sympatec GmbH, Clausthal-Zellerfeld, Germany), energy-dispersive X-ray fluorescence (XRF) spectrometry (EDX-7000 Shimadzu, Tokyo, Japan), and Fourier-transform infrared spectroscopy (FTIR) (Agilent Technologies, Palo Alto, CA, USA). All the operational conditions were identical to those used in our previous article [3].

### Antimicrobial activity testing

Distilled water was used to dilute the AgNPs to obtain a stock solution concentration of 1024 μg/mL. All stock solutions underwent sterilization of microfiltration (0.22 μm), and aliquots were diluted to create the various lower-concentration solutions required for the antimicrobial testing procedure. The antimicrobial activity of the AgNPs was assessed against the same Gram-negative bacteria used in a study by Arsene *et al*. [9]. These bacteria were: *Citrobacter freundii* 426, *Escherichia coli* 1449, *E. coli* ATCC 25922, *Achromobacter xylosoxidans* 4892, *Moraxella catarrhalis* 4222, *Pseudomonas aeruginosa* 3057, *Morganella morganii* 1543, and *Klebsiella oxytoca* 3003. Their full susceptibility profile to antibiotics is found in Table-1 of the study by Arsene *et al*. [9] (https://doi.org/10.3390/fermentation8110626). The well diffusion method was used to perform antibacterial testing, and the minimum inhibitory concentration (MIC) and minimum bactericidal concentration (MBC) were determined. The protocol was the same as that used by Arsene *et al*. [9] without any modification.

**Table-1:** The inhibition zone of the phytofabricated AgNPs using papaya root (AgNPs-PR) and papaya seed (AgNPs-PS) against selected Gram-negative bacteria.

Concentration (µg/mL)	Diameter of the inhibition zone (mm)							

	*A. xylosoxidans* 4892	*C. freundii* 426	*E. coli* 1449	*K. oxytoca* 3003	*M. catarrhalis* 4222	*M. morganii* 1543	*P. aeruginosa* 3057	*E. coli* ATCC 25922
AgNPs-PR								
200	16 ± 2	20 ± 1	8 ± 1	18 ± 2	26 ± 3	21 ± 2	16 ± 1	29 ± 3
100	11 ± 0	16 ± 1	0 ± 0	16 ± 1	21 ± 1	18 ± 0	9 ± 1	24 ± 1
50	9 ± 0	12 ± 0	0 ± 0	9 ± 0	18 ± 1	13 ± 2	6 ± 0	18 ± 0
25	6 ± 0	9 ± 0	0 ± 0	0 ± 0	15 ± 3	9 ± 0	0 ± 0	14 ± 1
5	0 ± 0	7 ± 0	0 ± 0	0 ± 0	7 ± 0	0 ± 0	0 ± 0	9 ± 0
AgNPs-PS								
200	11 ± 1	17 ± 3	0 ± 0	15 ± 2	19 ± 3	12 ± 1	11 ± 2	21 ± 1
100	6 ± 0	13 ± 1	0 ± 0	11 ± 1	15 ± 0	7 ± 0	7 ± 1	17 ± 3
50	0 ± 0	9 ± 0	0 ± 0	7 ± 0	11 ± 1	0 ± 0	0 ± 0	15 ± 1
25	0 ± 0	0 ± 0	0 ± 0	4 ± 0	6 ± 0	0 ± 0	0 ± 0	13 ± 0
5	0 ± 0	0 ± 0	0 ± 0	0 ± 0	0 ± 0	0 ± 0	0 ± 0	6 ± 0
Tetracycline (30 µg/disc)	6 ± 0	21 ± 1	24 ± 3	20 ± 1	12 ± 1	15 ± 0	6 ± 0	21 ± 3
Distilled water (sterile)	0 ± 0	0 ± 0	0 ± 0	0 ± 0	0 ± 0	0 ± 0	0 ± 0	0 ± 0

AgNPs=Silver nanoparticles, AgNPs-PR=Silver nanoparticles using papaya root, AgNPs-PS=Silver nanoparticles using papaya seed, *A. xylosoxidans=Achromobacter xylosoxidans, C. freundii=Citrobacter freundii, E. coli=Escherichia coli,* *K. oxytoca=Klebsiella oxytoca, M. catarrhalis=Moraxella catarrhalis, M. morganii=Morganella morganii,* *P. aeruginosa=Pseudomonas aeruginosa*

### Antibiofilm activity

As described in our previous study, the crystal violet attachment assay was used with slight modifications [8]. All experiments were performed in sterile 96-well microtiter plates. First, different mixtures (final volumes of 190 μL) consisting of brain heart infusion broth (BHIB) and AgNPs were prepared to achieve different concentrations of AgNPs (MIC/8, MIC/4, MIC/2, MIC, and 2MIC). Each concentration was placed into six wells (three for the test and three for the specific negative control). We used BHIB free of AgNPs as a negative control. The test wells were inoculated with 10 μL of an overnight culture of the test microorganism (18–24 h at 37°C and 1× *g*) and resuspended in sterile saline at 0.5 McFarland scale (approximately 1.5 × 10^8^ colony forming units/mL). After 48 h of incubation at 37°C, the medium was removed from the wells and replaced with 200 μL of 1% (w/v) crystal violet solution for 90 s. The wells were rinsed 3 times with distilled water before drying at 37°C. The biofilm-bound crystal violet was solubilized in 200 μL of 99% ethanol. The OD450 was measured and used to calculate the inhibition percentage.


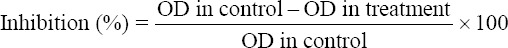


### Mechanism of antimicrobial activity

#### Action on growth kinetics

This test aimed at identifying the phase of the growth kinetics in which AgNPs exert antibacterial potential. The experiment used *E. coli* ATCC 25922, and the BHIB was mixed with the same concentrations as above (MIC/8, MIC/4, MIC/2, MIC, and 2MIC). Demgne *et al*. [35] suggested that the optical density at 450 nm (OD_450_) was measured at 0, 0.5, 1, 2, 4, 6, 8, 10, 12, 14, 24, and 48 h. Brain heart infusion broth free of AgNPs but inoculated with *E. coli* ATCC 25922 was used as a negative control. Optical density versus time curves were used to present the results.

### Action on H+ adenosine triphosphatase (ATP)ase-mediated proton pumping

This step, which was performed by tracking the acidification of the external medium by measuring the pH with a pH meter, showed the ability of synthesized AgNPs to inhibit *E. coli* ATCC 25922 H+-ATPase-mediated pump [35]. The pH evolution curve was shown as a function of time using the same concentration as at the previous section (Action on growth kinetics) and the recorded pH values (pH = f [time]). The H+-ATPase pumps have been implicated as the cause of any inhibition of the medium’s ability to become more acidic in the presence of AgNPs.

## Results and Discussion

### Biosynthesis and characterization of AgNPs

**Figure-1:**
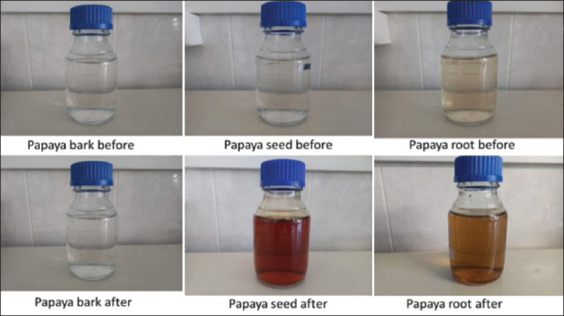
Coloration of the reaction mixture before and 24 h after synthesis of silver nanoparticles with papaya bark, root, and seed extracts.

The phytofabrication of AgNPs used an aqueous solution of the parts of *C. papaya* L. (seed, bark, and root) as reducing agents to reduce silver nitrate at 42°C and 5× *g*. The entire process was performed under low-light conditions to reduce the photo-activation effect of the Ag(I) ions. As shown in Figure-1, no color change was observed with papaya bark, whereas the AgNO_3_ solution changed from colorless to brown after adding PS and papaya root (PR) extracts. This first visual observation proved that the phytofabrication of the AgNPs occurred with PR (PR-AgNPs) and PS (PS-AgNPs) and confirmed that these plant extracts contained reducing and stabilizing agents, which reduced Ag+ to AgNPs. In addition, papaya bark extract does not contain phytochemicals capable of reducing Ag+, so it was not further considered in our investigation. Given that all plant samples were collected from the same papaya tree, it can be concluded that this difference is due to the compositional differences between the plant parts, exactly as suggested by Oliveira *et al*. [36]. Moreover, the reported color change (Figure-1) is characteristic of the formation of AgNPs and has been ascribed to the excitation of surface plasmon vibrations in AgNPs [37, 38].

**Figure-2:**
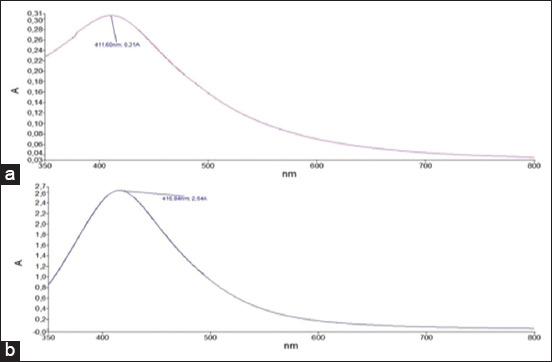
Ultraviolet–visible spectra of phytofabricated silver nanoparticles using (a) papaya root and (b) papaya seed.

**Figure-3:**
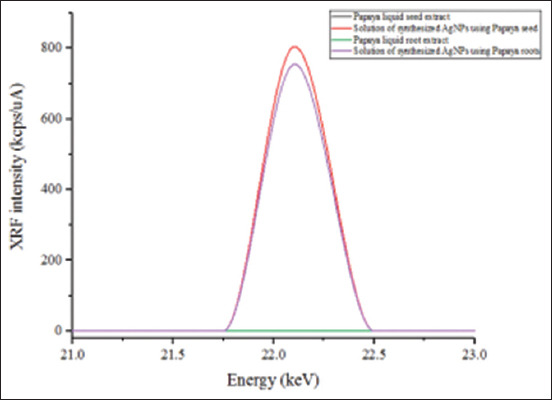
X-ray fluorescence spectra of papaya seed extract (black), solution of green-synthesized silver nanoparticles (AgNPs) using papaya seeds (red), papaya root extract (green), and solution of green-synthesized AgNPs using papaya roots (purple) at the characteristic energy of silver fluorescence - silver Kα line of 22.105 keV. The black and green lines coincide with the X-axis, showing that papaya seed and root extract does not contain Ag.

As shown in Figure-2, the UV-visible spectra of AgNPs mediated by PR and PS showed maximum absorption peak bands at 411.6 and 416.8 nm, respectively. However, the operating conditions were not the same. Similar wavelength results (416 nm and 411 nm) were obtained for cauliflower (*Brassica oleracea*) and orange (*Citrus sinensis*) peel extract-mediated synthesis of AgNPs [39, 40]. The absorbances at the optimal wavelengths were 2.6 and 0.3 A for PR-AgNPs and PS-AgNPs, respectively; these findings were in accordance with the visual observation (Figure-1). It has been reported that this difference in color and absorption is directly correlated with the concentration of AgNPs in the reaction mixture [39], verified by XRF in our study. Indeed, as shown in Figure-3, XRF confirmed the presence of Ag in both PR-AgNPs and PS-AgNPs by a characteristic peak in the spectrum at the silver Kα line of 22.105 keV (Figure-3). The fluorescence intensity turned out to be slightly higher in the solution of the synthesized NPs using PS (PS-AgNPs), which may indicate a higher silver concentration in PS-AgNPs compared to the synthesized AgNPs using PRs (PR-AgNPs).

**Figure-4:**
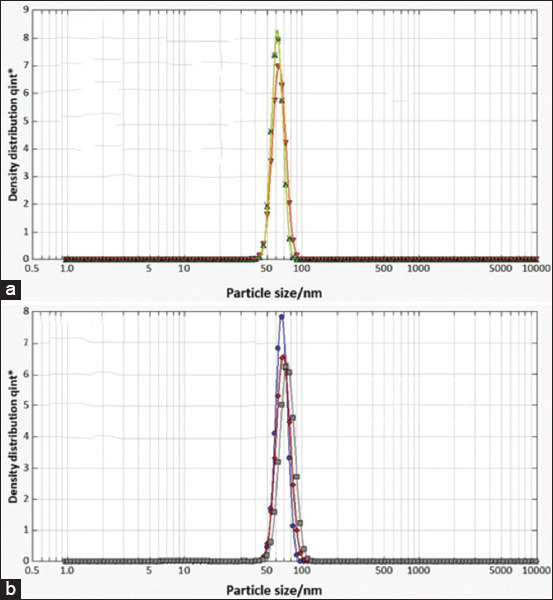
Hydrodynamic diameter of synthesized silver nanoparticles using (a) papaya root and (b) papaya seed.

**Figure-5:**
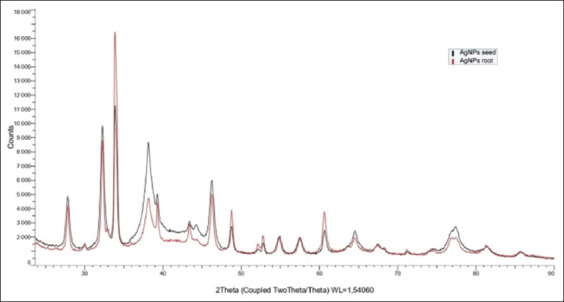
X-ray diffraction pattern of phytofabricated silver nanoparticles using papaya root and papaya seed.

In addition, the PCCS revealed that the X50 hydrodynamic diameters of PR-AgNPs and PS-AgNPs were 59.46 ± 7.03 and 66.57 ± 8.89 nm, respectively (Figure-4). Given that the AgNPs have diameters ranging from 0 to 100 nm [3, 17] and given the results presented above, it could be easily concluded that the PS and PR were successfully used to synthesize AgNPs in the present study. The crystalline nature of both AgNPs was confirmed by X-ray diffraction (XRD) as shown in Figure-5; the XRD pattern revealed Bragg reflections that indicated a face-centered cubic structure of Ag.

**Figure-6:**
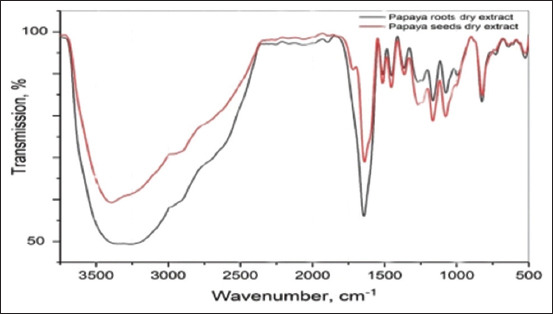
Fourier-transform infrared spectroscopy spectra of papaya root and papaya seed dried extracts.

**Figure-7:**
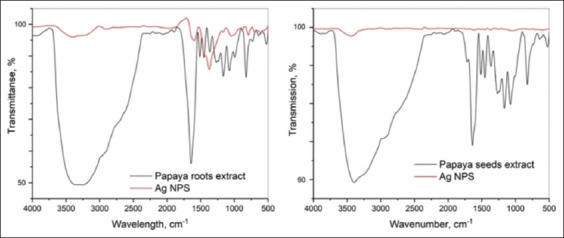
Fourier-transform infrared spectroscopy spectra of phytofabricated silver nanoparticles with the respective extracts used (papaya root and papaya seed).

Moreover, the FTIR spectra of PR and PS extracts are shown in Figure-6. Comparing the spectra of the two extracts, it was established that, in general, their chemical compositions were similar. Judging by the absorption bands, it can be decided that the extracts contain polyphenolic compounds. Indeed, a wide band of O–H stretching vibrations was observed in the region of 3000 cm^−1^–3500 cm^−1^, secondary and primary OH-group stretching vibrations at 1168 and 1081 cm^−1^, respectively, and phenolic hydroxyl bending vibrations at 1358 cm^−1^. The absorption band of the C–H group, which should be observed at 2900 cm^−1^–3000 cm^−1^, was hidden due to the strong influence of the O–H bond (the influence of the residual amount of water is not excluded from the study). However, methyl group bending vibrations were detected at 1451 cm^−1^. In addition, the band at 822 cm^−1^ is also characteristic of C–H bond deformation vibrations. A strong absorption band, presented as a doublet in the spectrum of the seed extract, in the region of 1710 cm^−1^–1650 cm^−1^ corresponded to the carbonyl group vibrations and probably indicates the presence of conjugated acids or aldehydes in the mixture. It is quite difficult to accurately judge the presence of amino derivatives; probably, the shoulder in the region of 2904 cm^−1^–2916 cm^−1^, which was more pronounced in the spectrum of the seed extract, are caused by the N-H group vibrations. A double band supports this assumption at 1256 cm^−1^ in both spectra, which is characteristic of the C–N bond stretching vibrations of an amine. In addition, Figure-7 shows the spectra of PS extract and PS-AgNPs. The technique used did not allow preservation of the stabilizing layer of organic components on the surface of the PS-AgNPs (Figure-7). In addition, the spectral analysis of AgNPs obtained using the PR extract showed the presence of stabilizing organic fragments containing C=O, C–H, O–H groups on the surface of the NPs (Figure-7).

### Inhibition zones of phytofabricated AgNPs

This study focused on using aqueous extracts of PS-AgNPs and (PR-AgNPs) to analyze the antibacterial potential of green-synthesized AgNPs against selected Gram-negative bacteria**.** Although the result is not presented here, it is important to clarify that we attempted to evaluate the inhibition zones (IZs) of the PR and PS extracts themselves, but no antimicrobial activity was observed. Notwithstanding this, the results of the IZ of the AgNPs at different concentrations (200, 100, 50, 25, 5, and 0 μg/mL) are recorded in Table-1. As expected, the negative control (distilled water used to prepare the solutions of AgNPs) showed no IZ (0 mm). Despite the bacterial strains, it was discovered that the inhibition diameters decreased with a decrease in the concentration of both AgNPs, which may indicate that the phytofabricated AgNPs are dose-dependent. Similar results have usually been reported in most studies investigating the antibacterial properties of antimicrobial compounds [3, 4]. At lower amounts of AgNPs-PS (5 μg/mL), all of the clinical strains showed resistance, but at the same concentration, an IZ of 7 ± 0 mm was found with AgNPs-PR against *C. freundii* 426. Comparatively, AgNPs-PS was less effective than AgNPs-PR against all the bacteria tested. By decreasing order, the most sensitive (highest IZs) bacteria to both AgNPs were *E. coli* ATCC 25922, *M. catarrhalis* 4222, *M. morganii* 1543, and *C. freundii* 426, whereas the most resistant was *E. coli* 1449. Interestingly, the sensitivity of bacteria to AgNPs correlated (p < 0.05) with their susceptibility to antibiotics and their multidrug resistance (MDR) index (Table-1 from https://doi.org/10.3390/fermentation8110626). Indeed, as demonstrated in Table-1, bacteria that exhibited the highest MDR index were more resistant than those with lower ones. This finding may be ascribed to the phenotypic resistance governed by the availability of the efflux pumps that are known to exist in Gram-negative bacteria. Besides the ability to produce antibiotic-degrading enzymes (i.e., β-lactamase genes), numerous Gram-negative bacteria have efflux pumps that expel antimicrobial molecules from cells, rendering the antibiotic ineffective [2]. Although the action mechanism of NPs has not yet been fully elucidated, the observations in the present study demonstrate that despite being sensitive when exposed to AgNPs, phenotypically, antibiotic-resistant bacteria have a better chance of survival than non-resistant bacteria.

### Minimum inhibitory concentration and MBC of green-synthesized AgNPs

**Figure-8:**
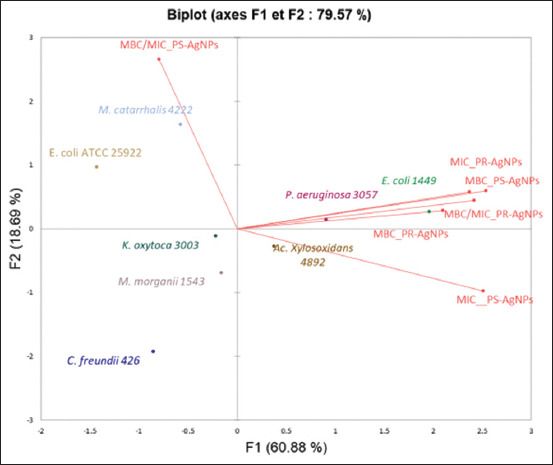
Principal component analysis analysis of minimum inhibitory concentration (MIC), minimum bactericidal concentration (MBC), and ratio MBC/MIC of phytofabricated AgNPs-PR and AgNPs-PS against selected Gram-negative bacteria. AgNPs-PR=Silver nanoparticles using papaya root, AgNPs-PS=Silver nanoparticles using papaya seed.

The well diffusion test was performed as a preliminary screening test for antibacterial activity as previously suggested by Oda *et al*. [40]; therefore, a further investigation was required to determine the antibacterial activity of AgNPs using MIC and MBC values. Here MIC is referred to as the lowest concentration of AgNPs that inhibited bacterial growth by serial dilution. As presented in Table-2, the MIC values of AgNPs against the chosen Gram-negative bacteria varied from 16 to 64 μg/mL for PS-AgNPs and from 4 to 64 μg/mL for PR-AgNPs. Regarding PS-AgNPs, the MIC was 16 μg/mL against *E. coli* ATCC 25922 and *M. catarrhalis* 4222; 32 μg/mL against *K. oxytoca* 3003; 64 μg/mL against *A. xylosoxidans* 4892, *M. morganii* 1543, and *P. aeruginosa* 3057; and 128 μg/mL against *E. coli* 1449. The MIC observed with PS-AgNPs were 2–4 times lower than those found with PR-AgNPs against the same bacteria (Table-2), and this confirmed the observation made with IZs that PR-AgNPs had greater antibacterial activity than PS-AgNPs. Moreover, the MBC was defined as the lowest concentration of AgNPs that totally kills the bacteria (no growth on an agar plate). In the present investigation, MBC ranged from 32 μg/mL–256 μg/mL for PS-AgNPs and from 4 μg/mL–128 μg/mL for PR-AgNPs. Interestingly, the MBC of PR-AgNPs was equal to the MIC in all of the bacteria tested except against *E. coli* 1449 against which MBC = 4 MIC. Given the MBC/MIC ratios, it became obvious that both AgNPs had bactericidal activity since the MBC/MIC ratios were ≤4 (MBC/MIC ≤4) [3]. Instead, a principal component analysis (PCA) was performed to illustrate the relationship between the microbial stains investigated, MIC, MBC, and the tolerance level (MBC/MIC ratio) of both AgNPs, and the result was allocated in an F1 × F2 system (Figure-8). The PCA showed that the first two principal components (F1 and F2) accounted for 60.88% and 18.69%, respectively, of the total variation and explained 79.57% of it. All the bacteria were distributed into three groups, and each of these groups seemed to correlate with antibiotic resistance (Figure-8 and Table-1 from Arsene *et al*. [9]).

**Table-2:** The MIC and the MBC of phytofabricated AgNPs-PR and AgNPs-PS against selected Gram-negative bacteria.

Bacterial strains	MIC (µg/mL)		MBC (µg/mL)		MBC/MIC	
		
	AgNPs-PS	AgNPs-PR	AgNPs-PS	AgNPs-PR	AgNPs-PS	AgNPs-PR
*A. xylosoxidans* 4892	64	16	128	16	2	1
*C. freundii* 426	32	8	32	8	1	1
*E. coli* 1449	128	32	256	128	2	4
*K. oxytoca* 3003	32	16	64	16	2	1
*M. catarrhalis* 4222	16	16	64	16	4	1
*M. morganii* 1543	64	8	128	8	2	1
*P. aeruginosa* 3057	64	64	128	64	2	1
*E. coli ATCC* 25922	16	4	64	4	4	1

MIC=Minimum inhibitory concentration, MBC=Minimum bactericidal concentration, AgNPs=Silver nanoparticles, AgNPs-PR=Silver nanoparticles using papaya root, AgNPs-PS=Silver nanoparticles using papaya seed, *A. xylosoxidans=Achromobacter xylosoxidans, C. freundii=Citrobacter freundii, E. coli=Escherichia coli,* *K. oxytoca=Klebsiella oxytoca, M. catarrhalis=Moraxella catarrhalis, M. morganii=Morganella morganii, P. aeruginosa=Pseudomonas aeruginosa*

The strong antimicrobial activity observed in this study, despite some bacteria being resistant to standard antibiotics, is consistent with previous studies employing AgNPs [41–45]. The MIC and MBC values from the previous studies showed the high significant variance. Consequently, comparison of results is extremely challenging; as explained by Loo *et al*. [41], the biosynthetic process of AgNPs, their physicochemical characteristics, the methods for evaluating antimicrobial activity, and above all, the bacteria tested, are factors that significantly influence the results.

### Antibiofilm potential of green-synthetized AgNPs

**Figure-9:**
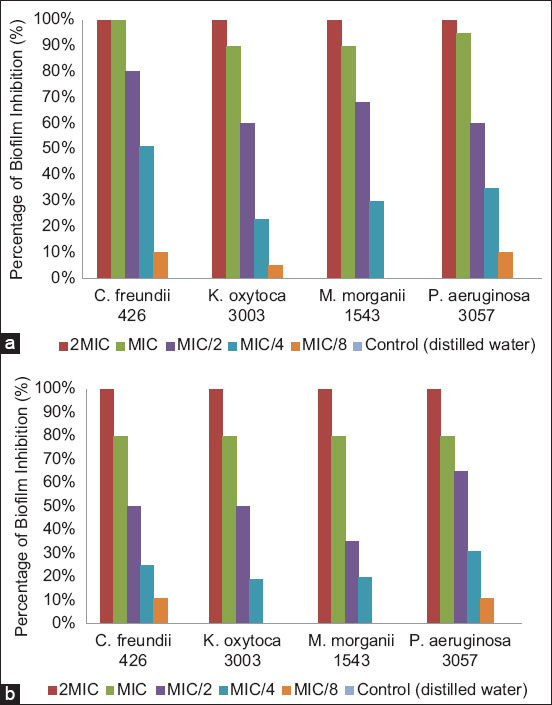
Antibiofilm activity of phytofabricated silver nanoparticles using (a) papaya root and (b) papaya seed.

Biofilm formation is a strategy used in bacteria consisting of a grouping in a matrix whose role in various infections can no longer be demonstrated [46]. This matrix can allow infections to significantly resist antibiotics, which has serious consequences in treating infections [3]. In the present study, we found that among the bacteria tested, only *P. aeruginosa* 3057, *K. oxytoca* 3003, *C. freundii* 426, and *M*. *morganii* 1543 were biofilm producers. After the antibiofilm activity investigation in a dose-dependent manner using the crystal violet attachment assay, we found that AgNPs strongly inhibited biofilm formation in all tested bacteria (Figure-9). As shown in Figure-9, treatment of all biofilm-forming bacteria at the specific 2MIC of AgNPs reduced biofilm formation by >99%. However, at the MIC, >99% biofilm inhibition was noticed only against *C. freundii* 426 (with PR-AgNPs), and inhibition percentage ranged from 70%–95% against the other bacteria with both AgNPs. Silver nanoparticles made from PR (PR-AgNPs) extract showed strong biofilm formation inhibition (>50%) at low concentrations (MIC/4). Compared with PR-AgNPs, higher concentrations (MIC/2 at least) of PS-AgNPs were required to reach a noteworthy biofilm inhibition. We showed that the AgNPs generated from the two papaya component extracts exhibited antibiofilm action against the tested bacteria at sub-inhibiting concentrations (MIC/8, MIC/4, and MIC/2). However, the antibiofilm activity of AgNPs has received a limited amount of study, but several other studies have reported findings similar to ours [47–49]. Both phytofabricated AgNPs could be recommended for managing anomalies in which biofilms are involved, given the limitations of this investigation.

### Mechanism of antibacterial activity

The antimicrobial potential of biogenic synthesized AgNPs has been increasingly investigated, but very little information exists in the literature on their mechanism of action. The present study aimed to investigate the mechanism of action of phytofabricated AgNPs employing PS-AgNPs and PR-AgNPs extracts by examining their effects on *E. coli* ATCC 25922 growth kinetics and H^+^-ATPase proton pumps.

**Figure-10:**
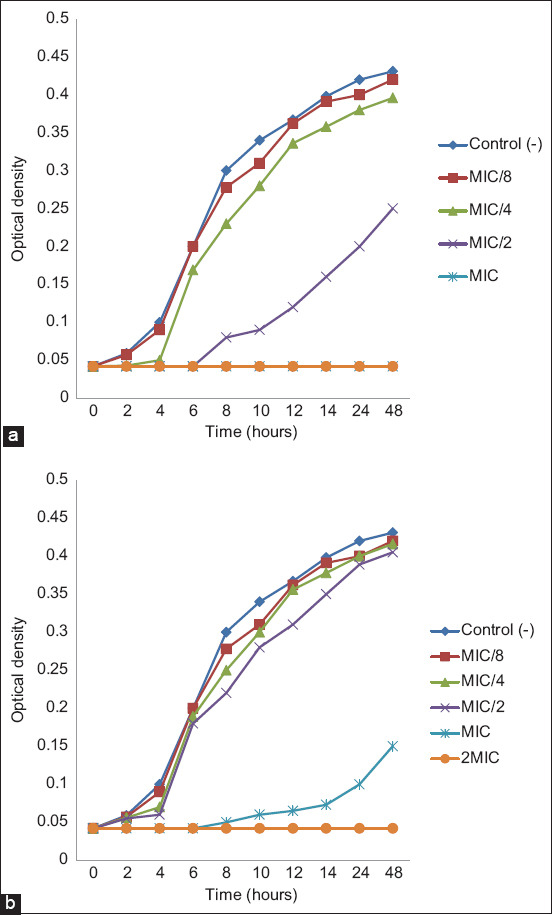
Action of phytofabricated silver nanoparticles using (a) papaya root and (b) papaya seed on *Escherichia coli* ATCC 25922 growth kinetic. MIC=Minimum inhibitory concentration.

**Figure-11:**
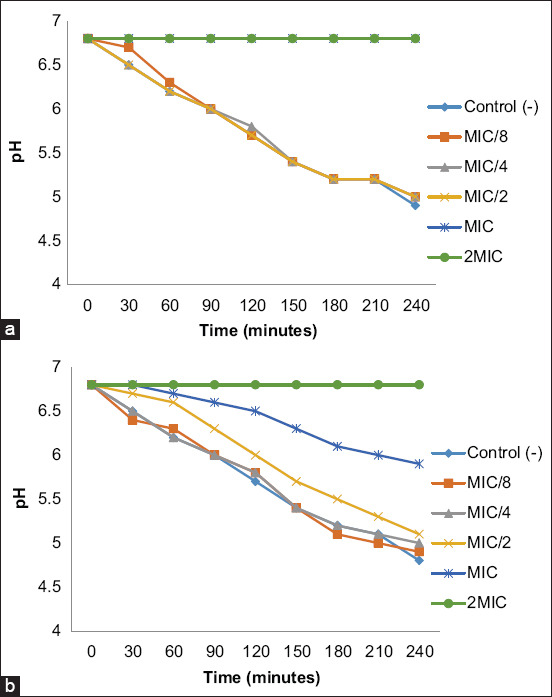
Action of phytofabricated silver nanoparticles using (a) papaya root and (b) papaya seed on *Escherichia coli* ATCC 25922 H^+^-ATPase-mediated proton pumping. MIC=Minimum inhibitory concentration.

As shown in Figure-10, at MIC/8 and MIC/4, both AgNPs did not influence *E. coli* growth. Surprisingly, this observation was inconsistent with the effect of PR-AgNPs MIC/4 concentrations on the biofilm formation (Figure-9). This result suggests that AgNPs can block the chemical signal involved in the bacterial cell-to-cell communication governed by the quorum-sensing system, thus preventing the bacteria from producing extracellular polymeric substances (EPS) and embedding together, without influencing bacterial growth. In addition, we found that at MIC/2, PR-AgNPs treatment extended the lag phase by ≤10 h while PS-AgNPs did not influence the growth kinetics. At MIC and 2MIC the lag phase was extended until the end (48 h) of the growth kinetic study with PR-AgNPs, whereas the lag phase was prolonged to 14 h with PS-AgNPs at the MIC and to the end with the PS-AgNPs at 2MIC. This result with PR-AgNPs could be explained by the fact that the MIC was equivalent to the MBC whereas with PS-AgNPs, the MBC was 4MIC. As expected, the same trend was observed in the effect of both AgNPs on *E. coli* H^+^-ATPase proton pumps (Figure-11). In fact, as shown in Figure-11, a gradual and significant increase in acidification of the media (pH decrease from 6.8 to 5.0) was noticed from 0 to 4 h for MIC/8, MIC/4 and the control. Unexpectedly, a slight pH decreases from 6.8 to 6 was observed at the MIC of PS-AgNPs. In contrast, no change in pH was observed at the PS-AgNPs 2MIC and MIC and the PR-AgNPs 2MIC. These results were attributed to the capacity of AgNPs to stop bacteria from using the nutrients present in the culture medium by inhibiting the creation of enzymes involved in nutrient metabolism. Any obstruction of this metabolic process could be detrimental to bacterial survival because the cytoplasmic pH of bacterial cells is controlled by protons extruding through the respiratory chain and K^+^ influx, and because ion-exchange systems in bacteria are linked to ATP energy synthesis. In addition, it can be hypothesized that at biocidal concentrations, given their tiny size (1 nm–100 nm) and as suggested by others [17, 40], AgNPs may readily adhere to the cytoplasmic membrane and cell wall to cause disruption and penetrate the cell, induce inactivation of cell proteins by silver ions, and possibly affect the production of reactive oxygen species that are also known to be harmful to bacteria.

## Conclusion

The study results indicated that AgNPs were successfully synthesized using PS and PR extracts and that papaya bark was not a good candidate for the phytofabrication of AgNPs. The average sizes of these phytofabricated nanostructures were 59.46 ± 7.03 nm for PR-AgNPs and 66.57 ± 8.89 for PS-AgNPs. The use of PS and PR extracts for phytofabrication of the AgNPs is fast, simple, and environmentally friendly, as demonstrated by UV-visible spectrophotometry, PCCS, XRD, energy-dispersive XRF, and FTIR. Both AgNPs showed noteworthy antimicrobial and antibiofilm activity in tests with specific Gram-negative bacteria. Given the good antimicrobial potential, further investigations are needed to assess toxicity before the synthesized AgNPs can be recommended for biomedical applications.

## Authors’ Contributions

MMJA, PIV, BZC, SLA and AKLD: Conceptualized and designed the study. MMJA, GOA, YNV, MA, MM, KP, BMN, VEA, BLA, ZAV, and VA: Conducted the laboratory experiments. MMJA, AKLD, BZC, GOA, YNV, and SLA: Data analysis and interpretation and drafted and revised the manuscript. All authors have read, reviewed, and approved the final manuscript.
